# Semaphorin-1a Is Required for *Aedes aegypti* Embryonic Nerve Cord Development

**DOI:** 10.1371/journal.pone.0021694

**Published:** 2011-06-27

**Authors:** Morgan Haugen, Ellen Flannery, Michael Tomchaney, Akio Mori, Susanta K. Behura, David W. Severson, Molly Duman-Scheel

**Affiliations:** 1 Department of Medical and Molecular Genetics, Indiana University School of Medicine, South Bend, Indiana, United States of America; 2 Department of Biological Sciences and Eck Institute for Global Health, University of Notre Dame, Notre Dame, Indiana, United States of America; VIB & Katholieke Universiteit Leuven, Belgium

## Abstract

Although mosquito genome projects have uncovered orthologues of many known developmental regulatory genes, extremely little is known about mosquito development. In this study, the role of *semaphorin-1a (sema1a)* was investigated during vector mosquito embryonic ventral nerve cord development. Expression of *sema1a* and the *plexin A (plexA)* receptor are detected in the embryonic ventral nerve cords of *Aedes aegypti* (dengue vector) and *Anopheles gambiae* (malaria vector), suggesting that Sema1a signaling may regulate mosquito nervous system development. Analysis of *sema1a* function was investigated through siRNA-mediated knockdown in *A. aegypti* embryos. Knockdown of *sema1a* during *A. aegypti* development results in a number of nerve cord phenotypes, including thinning, breakage, and occasional fusion of the longitudinal connectives, thin or absent commissures, and general distortion of the nerve cord. Although analysis of *Drosophila melanogaster sema1a* loss-of-function mutants uncovered many similar phenotypes, aspects of the longitudinal phenotypes differed between *D. melanogaster* and *A. aegypti*. The results of this investigation suggest that Sema1a is required for development of the insect ventral nerve cord, but that the developmental roles of this guidance molecule have diverged in dipteran insects.

## Introduction

Mosquito genome projects [1], [2], [3] have revealed orthologues of many genes that are known to regulate development of *D. melanogaster*, a well-characterized genetic model organism. Although characterization of the function of these mosquito genes could provide insight into the evolution of insect development or potentially reveal novel strategies for vector control, extremely little is known about mosquito development [4], [5]. Studying the development of mosquitoes has proven to be technically challenging, and although excellent descriptive analyses of *A. aegypti* embryogenesis exist [6], [7], expression of only a handful of mosquito embryonic genes has been described in *A. aegypti* or other vector mosquito species [8], [9], [10], [11], [12], [13], [14], [15], [16]. Given the importance of studying the biology of *A. aegypti* [4], [17], we recently published a series of protocols for analysis of the development of this mosquito [18], [19], [20], [21], [22]. These methodologies, in addition to those published previously [8], [10], are promoting analysis of mosquito developmental genetics.

A major research goal in our laboratory is to investigate the function of genes that regulate arthropod nervous system development. Analysis of genes that regulate mosquito nervous system development will promote a better understanding of the developmental basis of motor function, sensory processing, and behavior, key aspects of mosquito host location. The appearance of the mature ventral embryonic nerve cords of *A. aegypti*, *A. gambiae*, and *D. melanogaster*, as well as many other insects and crustaceans, are markedly similar [15], [16], [23]. Despite these similarities, differences in ventral nerve cord axonogenesis [15], [23] have been described. For example, in the case of the brine shrimp *Artemia franciscana*, the mechanism for pioneering the ventral nerve cord is different than what has been observed in *Drosophila* and other insects, and temporal changes in the expression of the axon guidance molecule Netrin parallel these differences [23]. Furthermore, although early axonogenesis in *A. aegypti* embryos is similar to that of *D. melanogaster*, our recent studies in this vector mosquito suggest that the function of Netrin signaling during embryonic ventral nerve cord development has evolved in insects [16]. siRNA-mediated knockdown of the *A. aegypti frazzled (Aae fra)* gene, which encodes a Netrin receptor, suggests that the developmental mechanisms responsible for regulating axon guidance in the embryonic nerve cord may have diverged among insects. Here, we examine the function of a second axon guidance molecule, Semaphorin-1a (Sema1a), in vector mosquitoes.

Sema1a, a member of the Semaphorin family of axon guidance molecules, is expressed in the developing central nervous system (CNS) of *Drosophila* [24]. During nervous system development, Semas guide neuronal growth cones by acting as attractants or repellents [25], [26]. In the fruit fly, Sema1a signals through the PlexA receptor and is critical for proper CNS formation and motor axon guidance, as evidenced by defasciculation abnormalities noted in mutant embryos [26], [27], [28]. Sema1a also has specific targeting functions in the developing *Drosophila* olfactory system [29], [30] and regulates photoreceptor axon guidance in the fruit fly visual system [31]. Furthermore, recent studies indicate that Sema1a can promote growth during *Drosophila* development through induction of key cellular growth regulators [32]. Orthologues of Sema1a were identified in both the *A. aegypti* and *A. gambiae* genome projects [1], [2], and it is hypothesized that Sema1a signaling will function in the development of the mosquito embryonic nervous system. In this investigation, we examine expression of *sema1a* in two mosquito species and investigate the impact of knocking down *sema1a* during *A. aegypti* embryonic nervous system development.

## Results and Discussion

### Expression of *sema1a* and *plexA* in vector mosquito embryos

**Figure 1:**
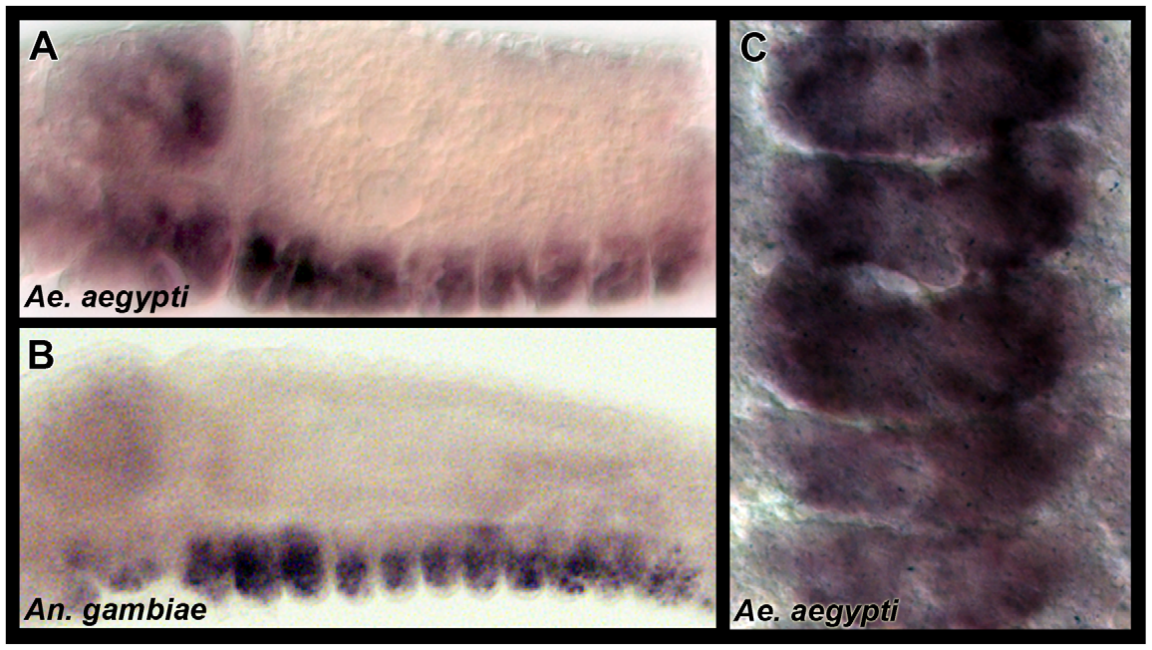
Expression of *sema1a* during vector mosquito development. *sema1a* expression is detected in lateral views of the developing nervous systems of *A. aegypti* (A, 54 hrs.) and *A. gambiae* (B, 33 hrs.). A ventral view of *Aae sema1a* expression (54 hrs.) is shown in C. Embryos are oriented anterior left/dorsal upwards in A and B and anterior upwards in C.

Orthologues of *sema1a* and *plexA* were identified in both the *A. aegypti* [2] and *A. gambiae* [1] genome projects. Orthology assignments were confirmed through phylogenetic analyses which are presented in Fig. S1. Expression of *A. aegypti sema1a (Aae sema1a)* and *A. gambiae sema1a (Aga sema1a)* were analyzed through whole-mount *in situ* hybridization at the onset of nerve cord development in both species. *Aae sema1a* expression initiates in most developing neurons of the CNS just prior to establishment of the axonal scaffold and is maintained during ventral nerve cord formation (Fig. 1A,C). Comparable *sema1a* expression patterns are detected in the developing nervous system of *A. gambiae* (Fig. 1B). A similar *sema1a* pattern of expression was previously reported in the developing CNS of *D. melanogaster* [24]. In *Drosophila*, Sema1a signals through the PlexA receptor [26], [27]. The expression patterns of *plexA* in both *A. aegypti* (Fig. S2A,C) and *A. gambiae* (Fig. S2B) are comparable to those of *sema1a* in both mosquito species (Fig. 1) and to the published patterns of *Drosophila plexA* expression [27]. These data are consistent with the hypothesis that Sema1a signaling regulates embryonic nervous system development in mosquitoes.

### siRNA-mediated knockdown of *sema1a* during *A. aegypti* development

We next functionally tested the hypothesis that *sema1a* expression is required for proper development of the ventral nerve cord in mosquitoes. This was accomplished through knockdown of the *A. aegypti sema1a* gene through use of RNAi, which was recently shown to be an effective method of inhibiting gene function during embryonic development of *A. aegypti* [16], [20]. Two siRNAs corresponding to different regions of the *Aae sema1a* gene, siRNA^890^ and siRNA^1198^, were used to target *sema1a*. Control experiments were completed using a control siRNA that has no sequence homology in the *A. aegypti* genome.

**Figure 2:**
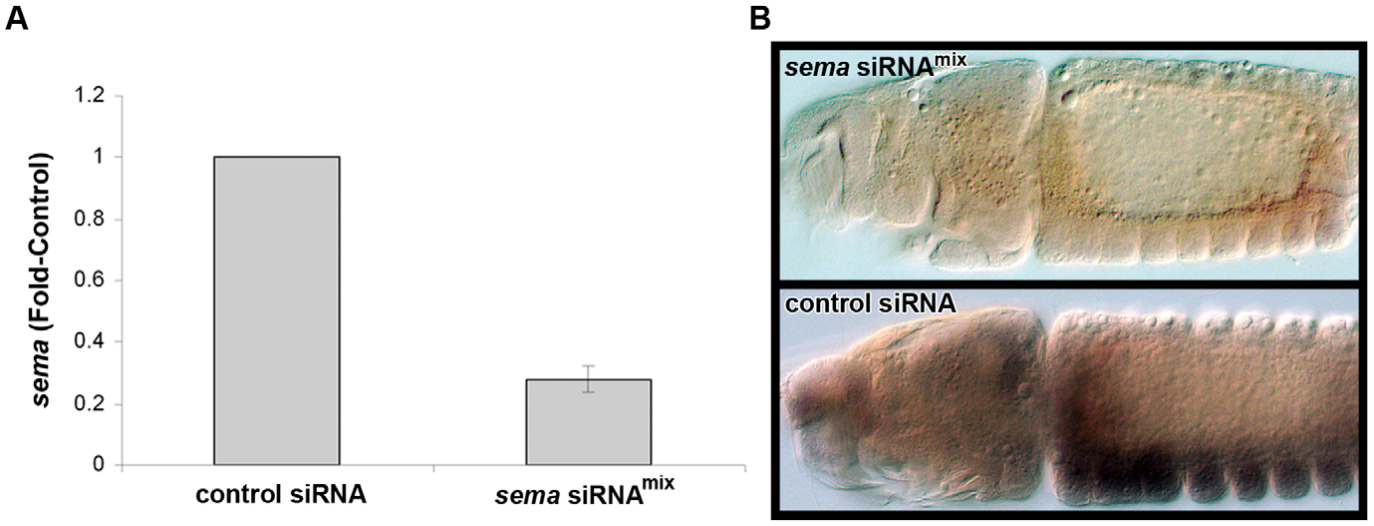
Confirmation of *sema1a* knockdown in *A. aegypti*. (A) qRT-PCR was used to assess *sema1a* levels 48 hrs. post-injection. *sema* mRNA levels were normalized to levels of the *RPS17* housekeeping gene. At 48 hrs. post injection of *sema* siRNA^mix^, *sema* transcript levels were on average 72% less than that of the control siRNA-injected group (p<0.01, N = 7, where N is the number of biological replicates). (B) Knockdown in the developing CNS (54 hrs.) was verified through *in situ* hybridization, which confirmed reduced levels of *sema1a* transcripts in the embryonic CNS at levels comparable to those detected by qRT-PCR, and which revealed nearly complete knockdown in a number of *sema* siRNA^mix^-injected embryos (upper panel, compare to control siRNA-injected embryo in lower panel). Embryos are oriented anterior to the left.

siRNAs were injected pre-cellular blastoderm, and *Aae sema1a* knockdown was confirmed through both quantitative real-time PCR (qRT-PCR) and whole-mount *in situ* hybridization. Multiple qRT-PCR replicates at three different time points, including 24, 48, and 72 hrs., confirmed knockdown throughout embryogenesis following injection of either siRNA^890^ or siRNA^1198^. Results are reported for 48 hrs. (Fig. 2A), a time point that corresponds to early neurogenesis. Injection of siRNA^890^ resulted in an average of 59% reduction of *sema1a* transcripts (N = 9, p<0.001, where N is the number of biological replicates), and injection of siRNA^1198^ resulted in an average of 63% reduction (N = 7, p<0.001) of *Aae sema1a* transcripts at 48 hrs. of development. siRNA^mix^, a combination of siRNA^890^ and siRNA^1198^, yielded the highest levels of knockdown. At 48 hrs. post injection of siRNA^mix^, *sema* transcript levels were on average 72% less than that of the control-injected group (N = 7, p<0.01, Fig. 2A). A maximum average of 83% knockdown was achieved in one siRNA^mix^ replicate.

Knockdown in the developing CNS was also verified through *in situ* hybridization at 54 hrs. of development, immediately preceding the time point at which axon phenotypes were assessed (see below). *In situs* confirmed reduced levels of *sema1a* transcripts in siRNA^mix^-injected embryos at levels comparable to those detected by qRT-PCR, and which revealed nearly complete knockdown in a number of siRNA^mix^-injected embryos (Fig. 2B).

### Analysis of the *A. aegypti sema1a* knockdown CNS phenotype

**Figure 3:**
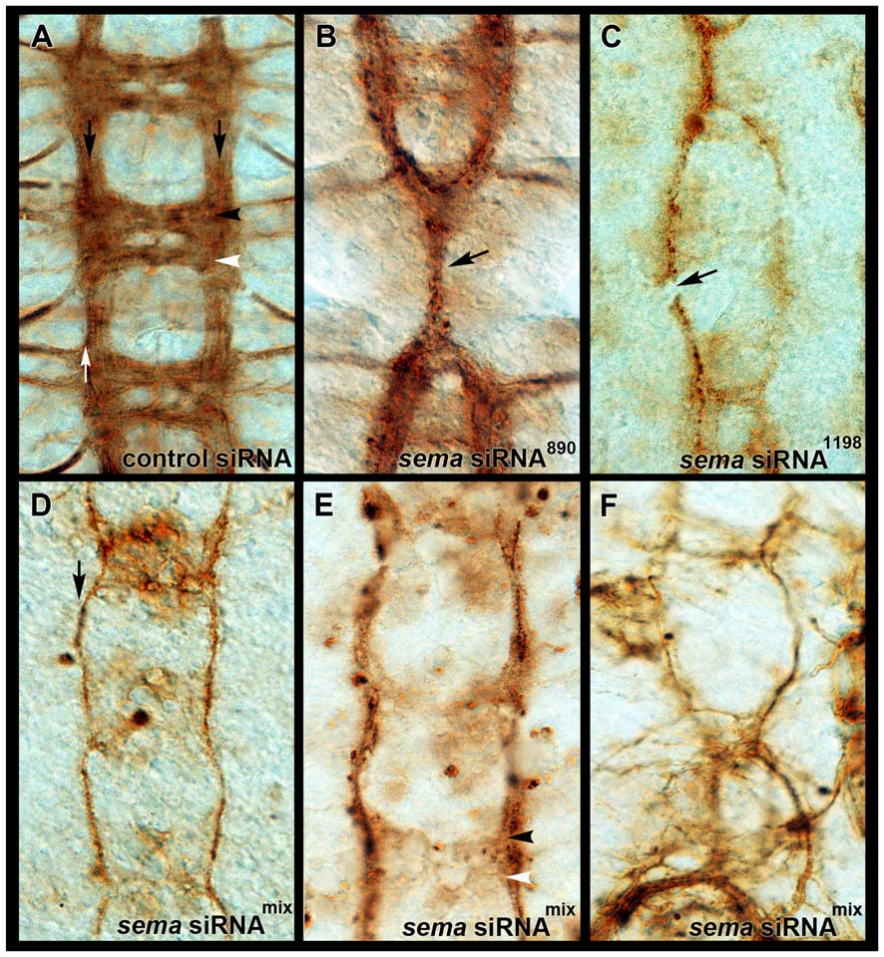
*A. aegypti sema1a* knockdown embryonic CNS phenotypes. Anti-acetylated tubulin staining marks the axons of the ventral nerve cords at 56 hrs. of development post-injection of siRNA-control injected (A) and *sema* siRNA-injected (B–F) embryos. Control-injected embryos had a wild-type appearance (A; longitudinals are marked by black arrows, while the third (outermost) fascicle of the left longitudinal connective is marked by a white arrow; the anterior commissure is marked by a black arrowhead, and the posterior commissure is marked by a white arrowhead). *sema* siRNA-injected embryos (B–F) were injected with different siRNAs/combinations of siRNAs targeting *sema1a*. These included: siRNA^890^ (B), siRNA^1198^ (C), or a combination of the two (siRNA^mix^, D–F). A number of phenotypes were observed in the *sema* siRNA-injected embryos, including thinning (C–F; marked by arrow in D), breakage (C–F; marked by arrow in C), or fusion (B,C,F; marked by arrow in B) of the longitudinals, thin or missing commissures (B–E; marked by arrowheads in E), and severe distortion of the nerve cord (F). Injection of either siRNA alone (B,C) or a combination of the two siRNAs (siRNA^mix^, D–F) generated comparable axon phenotypes. Filleted embryonic nerve cords are oriented anterior upwards in all panels.

**Figure 4:**
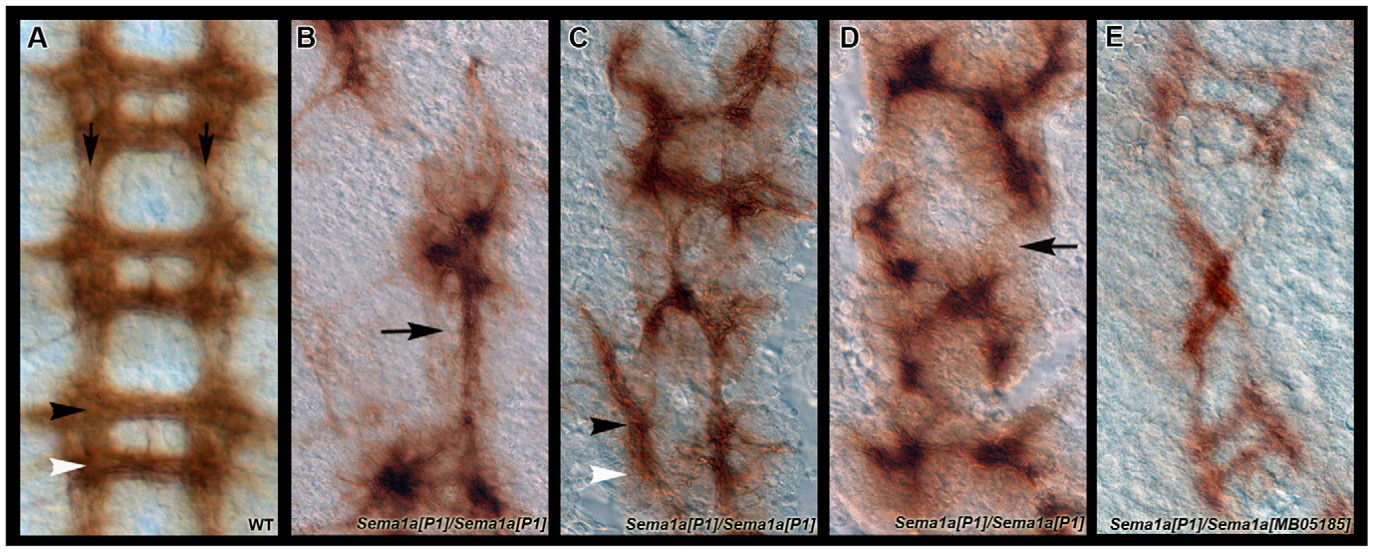
*D. melanogaster sema1a* loss of function embryonic CNS phenotypes. BP102 staining (A–E) labels axons of the embryonic CNS (wild-type in A; longitudinals are marked by arrows; the anterior commissure is marked by a black arrowhead, and the posterior commissure is marked by a white arrowhead). A variety of nerve cord phenotypes are observed in *sema1a[P1]* null mutants (B–D), including breakage (B–D; arrow in D) and occasional fusion (B,C,E; arrow in B) of the longitudinal connectives, thin or absent commissures (B–D; arrowheads in C), and general distortion of the nerve cord (B–D). Comparable phenotypes were observed in *Sema1a[P1]/Sema1a[MB05185]* heterozygotes (E). Filleted embryonic nerve cords are oriented anterior upwards in all panels.

In both *A. aegypti* and *D. melanogaster*, a scaffold of developing axon pathways give rise to the embryonic ventral nerve cord, which has a ladder-like appearance (Fig. 3A, Fig. 4A). A pair of bilaterally symmetrical longitudinal axon tracts are pioneered separately on either side of the midline in each segment. Although a fraction of early growth cones project only on their own side, most CNS interneurons project their axons across the midline in either the anterior or posterior commissural axon tracts before extending rostrally or caudally in the developing longitudinals [33], [34]. At 56 hrs. of development, the mature ventral embryonic nerve cord of *A. aegypti* (Fig. 3A) resembles that of the mature ventral nerve cord found in a St. 16 *D. melanogaster* embryo (Fig. 4A, [16]).

The impact of *sema1a* knockdown on *A. aegypti* embryonic nerve cord development was assessed through anti-acetylated tubulin staining at 56 hrs. of development. *sema1a* siRNA-injected embryos display a variety of nerve cord defects, including thinning, breakage, or fusion of the longitudinals (Fig. 3B–F), as well as thin (Fig. 3B–D) or missing (Fig. 3D–F) commissures. In some cases, the nerve cord was very severely distorted, almost appearing to be twisted upon itself (Fig. 3F). Injection of either siRNA independently (Fig. 3B and C) or a mixture of the two (siRNA^mix^, Fig. 3D–F) generated comparable axon phenotypes. Generation of the *sema1a* knockdown nerve cord phenotype with two separate siRNAs corresponding to different regions of the *sema1a* gene suggests that these phenotypes result from knockdown of *sema1a* and do not result from off-site targeting.

Although anti-Fasciclin II (Fas II) staining previously revealed motor neuron defects in *D. melanogaster* [26], attempts to score such phenotypes in *A. aegypti* were abandoned for a number of reasons. First, markers for particular motor neurons, i.e. anti-Fas II, are not yet available in *A. aegypti*. While the *Drosophila* anti-Fas II antibody has been useful in the characterization of fruit fly *sema1a* mutants [26], the *Drosophila* antibody does not crossreact with *A. aegypti* Fas II (not shown). Secondly, due to the severe nature of the midline phenotypes observed in *A. aegypti* (Fig. 3), it would be difficult to rule out that any motor neuron phenotypes observed were not simply secondary to defects at the midline.

### Comparative analysis of *sema1a* loss in *D. melanogaster*

We wished to assess if *Drosophila sema1a* loss of function mutant embryos exhibit ventral nerve cord pathfinding defects comparable to those observed in *A. aegypti*. Yu *et al.* [26] previously used Fas II staining to uncover defects in the third longitudinal connective of *sema1a[P1]* null mutants, which were reported to have a thin, discontinuous, and wavy appearance. We further assessed the *sema1a[P1]* loss of function phenotype through BP102 staining (which labels all axons of the CNS [35]) in stage 16 germ-band retracted embryos, a time point that corresponds to the developmental time at which *A. aegypti sema1a* knockdown phenotypes were assessed ([4], Fig. 3). This staining revealed that *Sema1a[P1]* null loss of function mutant embryos exhibit many of the nerve cord defects observed in the *A. aegypti sema1a* knockdown experiments. These include breakage and occasional fusion of the longitudinal connectives, thin or absent commissures, and general distortion of the nerve cord (Fig. 4B–D). Although *sema1a[P1]* is a null allele [26], the range of severity of the phenotypes observed varied just as it did in the mosquito knockdown experiments. Such variation is often observed in conjunction with null alleles for other axon guidance genes, such as *Netrin* or *frazzled*, and is typically attributed to the notion that the multiple guidance molecules regulating axonogenesis can compensate for each other [36], [37], [38].

In order to be certain that the defects associated with the *sema1a[P1]* allele were not specific to this fly stock, the *sema1a[P1]* allele was outcrossed to two additional *Sema1a Minos*-insertion alleles, *Sema1a[MB05185]* and *Sema1a[MB07938]* (both of which appear to be hypomorphic alleles, as they, unlike the null allele, are homozygous viable). Both *Sema1a[P1]/Sema1a[MB05185]* (Fig. 4E), and *Sema1a[P1]/Sema1a[MB07938]* (not shown) heterozygotes exhibited midline defects that were qualitatively similar to those observed in *Sema1a[P1]*/*Sema1a[P1]* null mutants. Such defects were not observed when the *Sema1a[P1]* allele was outcrossed to a wild-type strain (not shown).

A number of studies have characterized the molecular functions of Sema1a in the *Drosophila* motor and sensory systems. These studies may provide insight into the molecular roles of Sema1a in the fruit fly and mosquito embryonic nerve cord. Yu et al. [39] demonstrated through manipulation of Sema1a, Fas II, and Connectin levels that Sema1a mediates defasciculation in the motor system by functioning as a repellent. They concluded that proper axon guidance resulted from a balance between attractive and repulsive guidance cues. More recently, Zlatic et al. [40], who studied embryonic sensory neuron axons, demonstrated that Semas pattern the dorso-ventral axis in fruit fly embryos. They concluded that sensory axons are delivered to particular regions of the neuropile by their responses to systems of positional cues, including different levels and combinations of Semas in various dorso-ventral layers of the neuropile. Yu et al. [31] also demonstrated that reverse signaling of Sema1a, which interacts genetically with *Rho1*, regulates axon guidance in the *D. melanogaster* photoreceptor system. Finally, Sema1a is known to promote growth and induction of key growth regulators such as Myc and Cyclin D in flies, suggesting that it may promote axon growth in addition to guidance [32]. These studies suggest that Sema1a, through regulation of cell adhesion molecules such as Fas II, signaling molecules such as Rho1, and growth regulators such as Myc and Cyclin D, may control axon repulsion, attraction, defasciculation, growth, and/or dorso-ventral patterning in the fly and vector mosquito embryonic ventral nerve cord.

### Evolution of Sema1a function in insects

The results of this investigation suggest that Sema1a plays a critical role during development of both the *A. aegypti* and *D. melanogaster* ventral nerve cords. Many similar defects, including thinning or loss of the commissural axons and breaks or fusion of the longitudinals, were observed when Sema1a function was compromised in either species (Figs. 3,4). However, slight differences in the phenotypes, including thinning of the longitudinals in the mature nerve cord of *A. aegypti* (Fig. 3C–F) but not *D. melanogaster* (Fig. 4), were also observed. Likewise, defects specific to the third fascicle (Fig. 3A) previously observed in *D. melanogaster* [26] were not observed at a comparable time point in *A. aegypti* (Fig. 3B–F). These observations suggest that while the overall function of Sema1a in nerve cord development is largely conserved between *A. aegypti* and *D. melanogaster*, the developmental functions of this gene have diverged slightly between the two insects.

These results suggest that further analysis of embryonic nerve cord development in mosquitoes may uncover underlying differences between *D. melanogaster* and mosquito nervous system development. In support of this idea, our recent analysis of the *fra* knockdown phenotype in *A. aegypti* suggests that while this gene is required for commissural axon guidance in both mosquitoes and fruit flies, the penetrance and severity of the *A. aegypti* knockdown phenotype is greater than that of the *Drosophila fra* null mutant [16]. The combined results of these investigations indicate that characterizing the functions of additional axon guidance genes in *A. aegypti* and other insect species may uncover other differences in gene function. Although *sema1a* expression in *A. gambiae* is comparable to that of *A. aegypti* (Fig. 1), functional analysis of *Aga sema1a* would be necessary to verify if its developmental roles are conserved in different species of mosquitoes. This would require the application of siRNA-mediated embryonic knockdown strategies recently developed for *A. aegypti* [20] to *A. gambiae*, which is a future goal of this laboratory. In general, although temporal changes in axon guidance gene expression that reflect underlying differences in nerve cord development have been noted in crustaceans [23], the apparent divergence of axon guidance gene functions within dipterans is somewhat surprising given the many similarities in insect embryonic CNS development that had previously been reported (for example, see [41], [42]). However, as discussed previously [15], [23], there are numerous examples in which homologous nerve cords have been produced despite the fact that earlier developmental processes have diverged. Functional genetic analyses in mosquitoes and other emerging arthropod models may therefore continue to reveal functional differences of phylogenetically orthologous nervous system development genes. These functional differences might be minor, such as those reported in this investigation, or may perhaps be quite significant, especially when comparing the function of less closely related arthropod species.

In the future, we also hope to apply the *Aae sema1a* knockdown strategies employed in this investigation to later stages of development. In particular, it would be interesting to determine if Sema1a, which regulates axon targeting during *D. melanogaster* olfactory development [29], [30], functions in the developing olfactory systems of vector mosquitoes. It is important to extend comparative analysis of nervous system developmental genetics to the olfactory system, which has not been particularly well-analyzed in an evolutionary developmental genetic context in arthropods. Furthermore, the mosquito olfactory system is a tissue of particular interest to the vector community, as location of human hosts is an olfactory-driven behavior.

## Materials and Methods

### Ethics statement

This investigation was performed in accordance with the recommendations in the Guide for the Care and Use of Laboratory Animals of the National Institutes of Health. The animal use protocol (Study # 11-036) was approved by the University of Notre Dame Institutional Animal Care and Use Committee.

### Mosquito Rearing, Egg Collection, and Fixation

*A. aegypti* Liverpool-IB12 (LVP-IB12) and *A. gambiae* M Form mosquitoes were used in these investigations. Procedures for mosquito rearing and egg collection [21], [43] have been described previously. *A. aegypti* were fixed as described [19], and *A. gambiae* fixation was completed through use of a comparable procedure, except that eggs were fixed at room temperature.

### Drosophila Genetics

Embryos of the following genotypes were scored in this investigation: *Sema1a[P1]*/*Sema1a[P1]*, *Sema1a[P1]/Sema1a[MB05185]*, and *Sema1a[P1]/Sema1a[MB07938]*. *Sema1a[P1]* is a null allele [26] and was provided by A. Kolodkin. *Sema1a[MB05185]* (Bloomington Stock Center, #24243) and *Sema1a[MB07938]* (Bloomington Stock Center, #25579) were donated to the Bloomington Stock Center by H. Bellen; our analyses (see results) suggest that both of these alleles behave as hypomorphic loss of function mutations. Additional information about these strains is available at Flybase ([44], http://flybase.bio.indiana.edu).

### Immunohistochemistry

Immunohistochemistry was performed in mosquitoes as previously described [18]. *Drosophila* embryos were prepared and stained according to the Patel [35] protocol. Anti-acetylated tubulin (Zymed, San Francisco, CA) was used at a final concentration of 1∶100, and BP102 (supplied by N.H. Patel) was used at 1∶10. HRP-conjugated secondary antibodies (Jackson Immunoresearch, West Grove, PA) were used at a concentration of 1∶200.

### In situ hybridization

Riboprobes corresponding to the *Aae sema1a* (AAEL002653), *Aga sema1a* (AGAP008656), *Aae plexA* (AAEL002346), and *Aga plexA* (AAGAP000064) genes were synthesized according to the Patel [45] protocol. Additional information about these genes is available at Vectorbase ([46], http://www.vectorbase.org). *In situ* hybridization was performed as previously described [22].

### RNA interference

Knockdown was performed through embryonic microinjection of siRNAs targeting *Aae sema1a* (for transcript information, see AAEL002653 at http://www.vectorbase.org, [46]). siRNA design and microinjection were performed as previously described [20]. The following siRNAs were synthesized by Dharmacon RNAi Technologies (Lafayette, CO): siRNA^890^ sense: 5′ AUCGUUAGAACCAUGCAAUUUUU 3′ and antisense: 5′ UUUAGCAAUCUUGGUACGUUAAA 3′ (corresponds to base pairs 890–1,011 of *Aae sema1a*), siRNA^1198^ sense: 5′ GCAAGGUUACAGAGGUAUGUU 3′ and antisense: 5′ UUCGUUCCAAUGUCUCCAUAC 3′ (corresponds to base pairs 1,198–1,219 of *Aae sema1a*), and control siRNA sense: 5′ UUCAGACUCGCUGAACACGUUUU 3′ and antisense: 5′ UUAAGUCUGAGCGACUUGUGCAA 3.′ The control siRNA is a scrambled version of an siRNA targeting *Aae sema1a*; Blast searches confirmed that this scrambled sequence does not target other genes in the *A. aegypti* genome. siRNAs were injected at a concentration of 6–8 ug/uL.

Measurement of knockdown effectiveness was determined both through *in situ* hybridization (see above) and through qRT-PCR. qRT-PCR experiments were performed as previously described [47]. In summary, for each replicate total RNA was extracted from ∼30 pooled siRNA-microinjected mosquito embryos using Trizol (Invitrogen, Carlsbad, CA). cDNA was prepared with the High Capacity RNA to cDNA Kit (Applied Biosystems, Foster City, CA) per the manufacturer's instructions. Real-time quantification was performed through use of the SYBR Green I PCR kit (Applied Biosystems, Foster City, CA) in conjunction with an Applied BioSystems Step One Plus Real-Time PCR System. Primer sets for *Aae sema1a* were: For 5′ CGCTGATGGATGAAAATGTG 3′ and Rev 5′ CCCACCGGGAGTTTTAATTT 3′. Primer sets for the housekeeping gene *RPS17*, which was included as the internal standard for data normalization [47], are: For 5′ AGA CAA CTA CGT GCC GGA AG 3′ and Rev 5′ TTG GTG ACC TGG ACA ACG ATG 3′. At least seven independent biological replicates were conducted, and all PCR reactions were performed in triplicate. Quantification of results was made by standardizing reactions to *RPS17* levels and then using the ΔΔCt method as described [48]. Results were expressed as fold-difference as compared to control-injected embryos. The Student's t-test was used to analyze qRT-PCR data from replicate experiments.

### Phylogenetic analyses

The orthologues of *Drosophila sema* and *plex* genes in the mosquito genomes were identified from OrthoDB ([49], http://cegg.unige.ch/orthodb3/) and Biomart ([50], http://www.biomart.org/). The orthologous prediction of these genes was consistent between both databases. The amino acid sequences of Sema and Plex from mosquitoes and the twelve *Drosophila* species were obtained from Vectorbase ([46], www.vectorbase.org) and Flybase ([44], www.flyabse.org). Multiple sequence alignment was performed using ClustalW [51]. A phylogenetic tree was drawn using the Neighbor-Joining method [52]. The bootstrap consensus tree was inferred from 1000 replicates [53]. Tree linearization was performed using methodology in reference [54] and assuming equal evolutionary rates in all lineages. The evolutionary distances were computed using the Poisson correction method [55] and correspond to the number of amino acid substitutions per site. Phylogenetic analyses were conducted in MEGA4 [56].

## Supporting Information

Figure S1 **Phylogenetic relationships of** ***sema*** **and** ***plex*** **orthologues.** A Neighbor-Joining phylogenetic tree of mosquito and *Drosophila* Sema and Plex proteins, which share sequence similarity, is shown. The tree topology shows distinct clades of orthologous proteins. The color coding of phylogenetic groupings are: Red = Sema1a, Fuscia = Sema1b, Olive = Sema2a, Green = Sema5c, Aqua = PlexB, and Blue = PlexA. Bootstrap values greater than 50% (based on 1000 replicates) are shown on nodes. Corresponding gene accession numbers are provided for reference.
(TIF)

Figure S2 **Expression of** ***Aae plexA*** **during vector mosquito development.** *plexA* expression is detected in lateral views of the developing nervous systems of *A. aegypti* (A, 54 hrs.) and *A. gambiae* (B, 33 hrs.). A ventral view of *Aae plexA* expression (54 hrs.) is shown in C. Embryos are oriented anterior left/dorsal upwards in A and B and anterior upwards in C.
(TIF)
