# Supplementary figures and images for: Semaphorin-1a Is Required for Aedes aegypti Embryonic Nerve Cord Development
Source: PLoS One. 2011 Jun 27;6(6):e21694. doi: 10.1371/journal.pone.0021694 (PMC3124551; doi:10.1371/journal.pone.0021694)

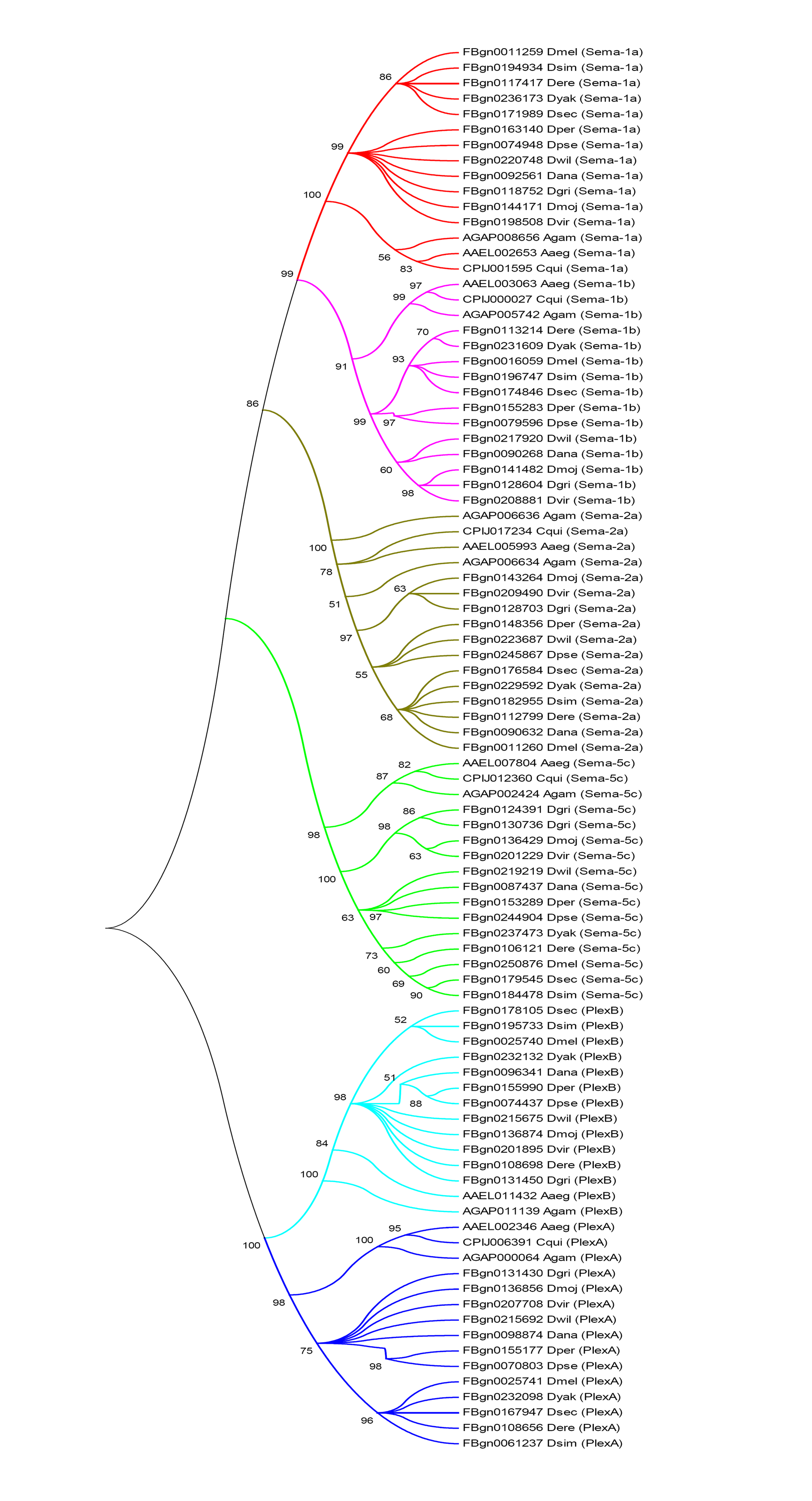

Supplement: Figure S1 — Phylogenetic relationships of sema and plex orthologues. A Neighbor-Joining phylogenetic tree of mosquito and Drosophila Sema and Plex proteins, which share sequence similarity, is shown. The tree topology shows distinct clades of orthologous proteins. The color coding of phylogenetic groupings are: Red = Sema1a, Fuscia = Sema1b, Olive = Sema2a, Green = Sema5c, Aqua = PlexB, and Blue = PlexA. Bootstrap values greater than 50% (based on 1000 replicates) are shown on nodes. Corresponding gene accession numbers are provided for reference. (TIF) [file pone.0021694.s001.tif]

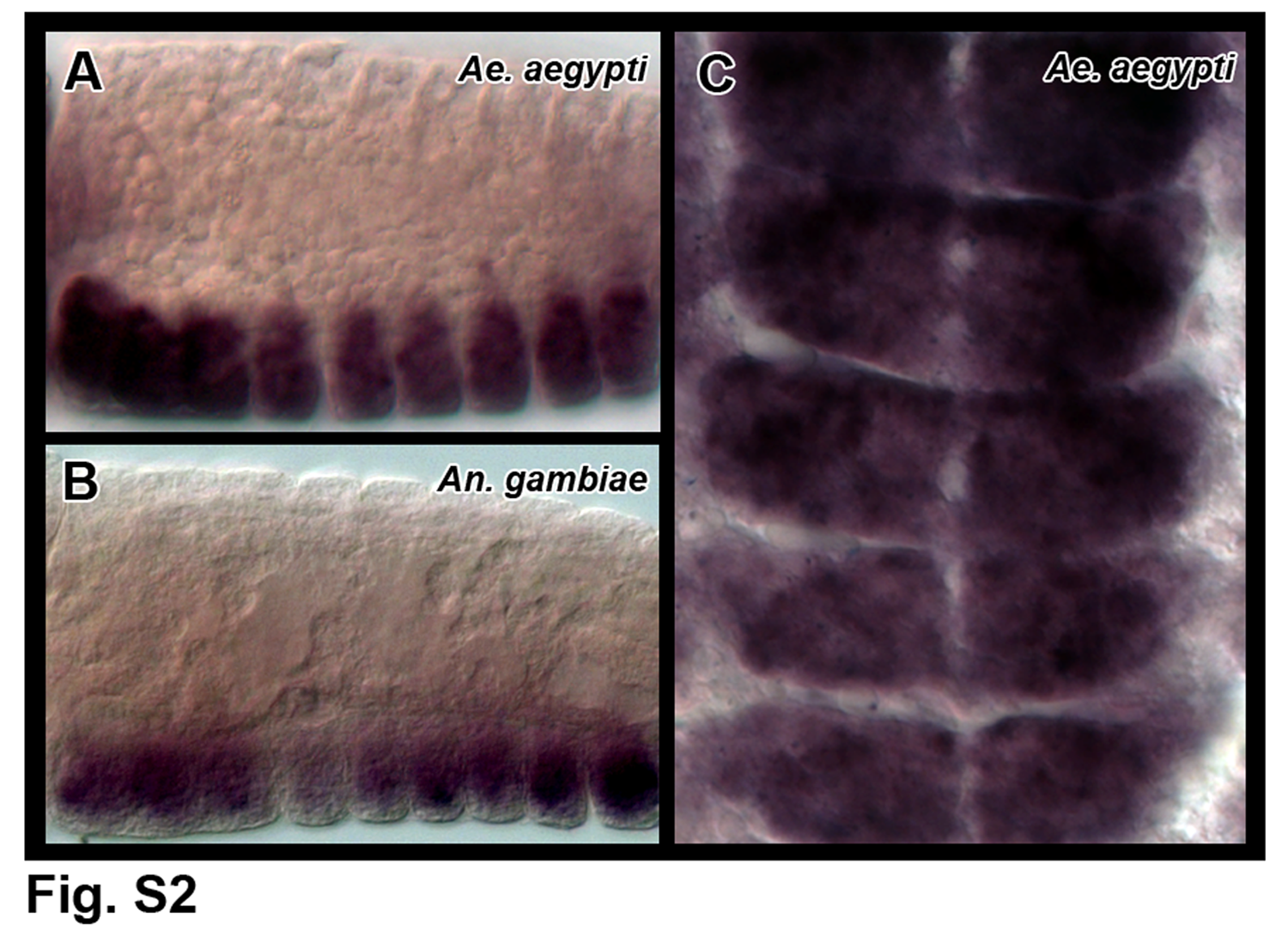

Supplement: Figure S2 — Expression of Aae plexA during vector mosquito development. plexA expression is detected in lateral views of the developing nervous systems of A. aegypti (A, 54 hrs.) and A. gambiae (B, 33 hrs.). A ventral view of Aae plexA expression (54 hrs.) is shown in C. Embryos are oriented anterior left/dorsal upwards in A and B and anterior upwards in C. (TIF) [file pone.0021694.s002.tif]
